# From Mandatory School Gymnastics to Physical Training for Youth. How the *Société Fédérale de Gymnastique* Became a “Gymnastic State” Dedicated to the Physical Preparation of Swiss Youth From 1873 to 1907

**DOI:** 10.3389/fspor.2021.563324

**Published:** 2021-02-26

**Authors:** Gil Mayencourt, Grégory Quin

**Affiliations:** Institut des Sciences du Sport de l'Université de Lausanne, Université de Lausanne, Lausanne, Switzerland

**Keywords:** gymnastics, physical education, Switzerland, youth, nation-building

## Abstract

The aim of this contribution is to analyse the special role that gymnastics clubs played, under the umbrella of the *Société Fédérale de Gymnastique* (SFG), in the formation of the Swiss nation and in the representation of a strong and united national youth at a time when the unity represented by the Swiss federal State founded in 1848 was strongly questioned by the conservative opposition. The purpose is mainly based on extensive statistical data gathered within the SFG about its members throughout the country at three particular moments (1873, 1895, and 1907) and on institutional archival funds. Our analysis is based on three successive points: after defining the relationship between the SFG and the Swiss Federal State (founded in 1848) for the unification and defense of the homeland, whether in terms of institutional mimicry or the building of a “national youth,” a second part defines which type of “youth” is specifically targeted by the SFG and what it meant at the time to be a “young” or an “old” gymnast, in particular through the participation of the SFG in the National exhibition of Geneva in 1896. Finally, a last part widens the perspective by highlighting, on one side, the cultural, political, and cantonal constraints on national expansion through youth of Swiss gymnastics and, on the other side, how these constraints have generated unifying and patriotic ambitions and discourses within the SFG.

## Introduction

From as early as the beginning of the nineteenth century, Swiss associations prepared the ground for the political and social processes that resulted in the Switzerland of 1848 (Jost, 1991; Humair, 2009). Thus, the foundation of the *Société Fédérale de Gymnastique* (SFG) in 1832 anticipated and announced, both with the use of the term “federal” and by the “supracantonal” structure it established, the installation of the federal State: the “modern Switzerland.”

Indeed, the institutionalization of Swiss gymnastics at the federal level happened 16 years before a countrywide and centralized political structure was established by the liberal-radical stream, which gradually took precedence over the conservative power from the 1830's onwards (Zimmer, 2003). The new federal State of 1848 was henceforth regulated by a national constitution which, among other things, united and governed all the Swiss cantons, even though these multi-faceted entities (with varying degrees of industrialization and urbanization, which are multilingual and multi-faith, and whose topography ranges between plains and mountains) retain part of their sovereignty through a tiered political system called “federalism.” Thus, it should be noted that the associationist trend—which can be defined as a regular meeting of people who follow a common goal and which is governed by free participation, equality among members and a general interest (Jost, 1991, p. 10)—marked the first steps of a common consciousness in a group of confederated cantons that wasn't yet “modern Switzerland.” In addition, the institutional operation of the associations has introduced very concrete ways of governing the group and allowing everyone's voice—even minority ones—to be heard.

In this process, the first gymnastics clubs appeared in German-speaking Swiss cantons in the first decades of the nineteenth century (1816 in Bern, 1819 in Basel and 1820 in Zurich) and the SFG was created on the occasion of what came to be recognized as the first “federal” gymnastics festival (Niggeler, 1882, p. 4–7). Organized in 1832 in Aarau by clubs from Aarau, Bern, Basel, Zurich and Luzern, it aimed to bring together all the local clubs in the country. The SFG, together with the *Société Suisse des Carabiniers* (1824)—the first genuinely “Swiss” association built around physical exercise—and the *Société Fédérale de Chant* (1842), opened the door of associationism to a wider section of the population, so that it was then no longer reserved for the elites alone (Wimmer, 2011, p. 726). In the specific case of the SFG, this democratization goes hand in hand with a strong ambition to target young people and to be a place for youth to experience the nation together.

To date, Swiss gymnastics has not really received very much attention from historians, and there is a huge lack of knowledge around this specific discipline in the field of physical and sports activities, especially in comparison with football or even skiing, first because it is the most popular sport in the country (Berthoud et al., 2016; Vonnard and Quin, 2019) and next because of its link with the tourism industry (Tissot, 2017). Actually, neither the national institution—the SFG—nor the gymnasts nor the leaders themselves have been the focus of any major research project (Vonnard et al., 2019). Nevertheless, several works have been published about the gymnastics festivals and their impact on the process of nation building (Schader, 1992; Schader and Leimgruber, 1993; Triet and Schildknecht, 2002), and we should also mention works on women's gymnastics, by Herzog (1995) and Quin (2015), which also tackled institutional processes. Similarly, gymnastics-based physical exercise was included in several research projects on physical education in schools from the nineteenth century onwards, thus directly related to the issue of youth (Burgener, 1952; Marcacci, 2000; Giuliani, 2001; Bussard, 2007; Czáka, 2008; Brühwiler, 2017; Horlacher, 2017). However, the gymnastics federal institution and its affiliated clubs, which are the main objects of this paper, were not at the core of any of these discussions.

With this in mind, several studies linking gymnastics clubs, youth and nation building carried out particularly in France and in Germany are inspirational and can definitely be seen as real resources for our analysis (Mosse, 1980; Arnaud, 1987; Defrance, 1987; Krüger, 1996). Furthermore, we will see that, based on a large sample of empirical data and through an approach focused on the territorial distribution of gymnastics clubs, our arguments corroborate the hypothesis already advanced in the literature that gymnastics in nineteenth century Switzerland was above all “an essentially urban, industrial and Protestant phenomenon.” (Marcacci, 2019, p. 141).

Parallel to that, youth history has only really emerged in the last couple of years, and although we can cite some general “women's history” or “workers history,” “youth” as a category has never really been studied from a historical point of view. The recent book by Bühler (2019) opens several new perspectives on the Swiss case, and points to references from abroad such as Marwick (1998) or Bantigny and Jablonka (2009), that might help us to think about the category, but also about the pre-First World War moment with Thiercé (1999) or also in Germany with several studies conducted around the “Wandervogel movement” (Klotter and Beckenbach, 2012). Alongside those references, considering “youth” as a specific group within society also raises questions about the “definition” and the “boundaries” of the group itself, especially at a time—around 1900—when life expectancy at birth was around 50 years (Floris et al., 2019, p. 220), not least because of the high infant mortality rate, and when reaching the age of 20 meant already having lived more than a third of one's life.

With this contribution, our aim is to underline the special role that gymnastics clubs played in the formation of the Swiss nation and in the representation of a strong and united national youth, at a time when a so-called centralism of the federal State was strongly questioned by the conservative opposition, but also sometimes in several French-speaking cantons (Humair, 2004). To do this, we undertake a combined analysis of the nationalization of Swiss gymnastics, including instances of resistance toward this process, and of the SFG's relationship with the “youth” group from the 1870's to the 1910's, keeping in mind that this moment was decisive for the institutionalization of the gymnastics movement (Jost, 1991) and for the introduction of mandatory gymnastics lessons in school (Burgener, 1952; Bussard, 2007). Indeed, instituted officially in 1874, on the ashes of the Sadowa battle and because of the threat caused by the Prussian army (Burgener, 1952, p. 81), the process of making gymnastics mandatory at school (for ages 10–16 years old), relied on the involvement of the gymnastics circles, both in order to train the newly required teachers and to offer courses for schoolboys between 16 and 20, to ensure that they remained fit for military service (Jaun, 1999). Also knowing that this was framed by the introduction of a mandatory primary school, often also influenced by the idea that the Prussian victories were those of the elementary school teachers (Westberg et al., 2019).

Our analysis is based on official documentation from the SFG (minutes, annual reports, and official bulletins—notably *Le Gymnaste* and *Schweizerische Turnzeitung*), but we also want to draw on extensive statistical data, compiled within the organization about its members all over the country, at three particular moments: 1873, 1895, and 1907. If these data sets are not always based on exactly the same framework, because they are compiled by different central committees, nevertheless all three address the spread of the practice in the country and the “age” of the gymnasts. To control the data provided in the three sets, we went through a systematic cross-referencing scheme, using the data sets and looking in the meantime into the annual reports and the minutes of the central committees where, year by year, meeting after meeting, data are compiled, compared and discussed within the gymnastics' elite. With the same ambition of cross-referencing data, we must mention some commemorative books written for the 50, 75, and 100^th^ anniversary, which are often quite subjective in their interpretation, but which can also address very precise contexts, with eye-witness accounts. In this idea, through the use of institutional archives, we have always compared published data (in commemorative books in particular) with the first data collection documents (handwritten tables sent by the member clubs for example), to ensure that there is no discrepancy between what is collected and what is then published. Having found no gap between these two types of sources, we included the data compiled in the handwritten tables in our research method and also supplemented them with official information published by the SFG. It should be noted that we are adding to this quantitative analysis a “discourse analysis” (Oger, 2005). Thus, we consider the published writings of the gymnastics' elite in official bulletins and commemorative books as characteristic of the ideological aims of their institution (SFG) that “configure and orient the production of discourse [written or oral] and the meaning given to activities within it” (115). This “discourse analysis” allows us to highlight the processes leading to the institutionalization of gymnastics, as a (sub)field influenced by politics, religion or even some economic dynamics (Quin, 2011; Vonnard and Quin, 2019).

After an introductory chapter that aims to define the relationship between the SFG and the federal State for the unification and defense of the homeland, whether in terms of institutional mimicry or the building of a “national youth,” the second part will define which type of “youth” is specifically targeted by the SFG and what it meant at the time to be a “young” or an “old” gymnast, in particular through the participation of the SFG in the National Exhibition of Geneva in 1896. Finally, the third part will widen the perspective by highlighting, on one side, the cultural, political and cantonal constraints on national expansion through youth of Swiss gymnastics and, on the other side, how these constraints have generated unifying and patriotic ambitions and discourses within the SFG.

## The *Société Fédérale De Gymnastique*: An Association Dedicated To Build A “Swiss National Youth”

A look at the official 1848 statutes of the SFG highlights the close links between gymnastics, patriotism and youth at the very moment when the unity of modern Switzerland was being formed. According to its statutes, the aim of the SFG is to “encourage body exercises among the Swiss people, to thereby enable them to bear arms for the defense of their homeland, and to unite young Swiss people through bonds of friendship and patriotic feelings” (SFG, Statutes, La Chaud-de-Fonds : 1848, p. 1). It was therefore a matter of preparing young people physically and ideologically for the defense of the homeland, but also of uniting them around the new liberal Swiss federal State created in the same year. From 1848 onwards, the interaction between the State and the SFG grew and a mirror effect was created by the gymnasts during the second half of the nineteenth century.

### Between Mimicry and Parastatism

**Figure 1:**
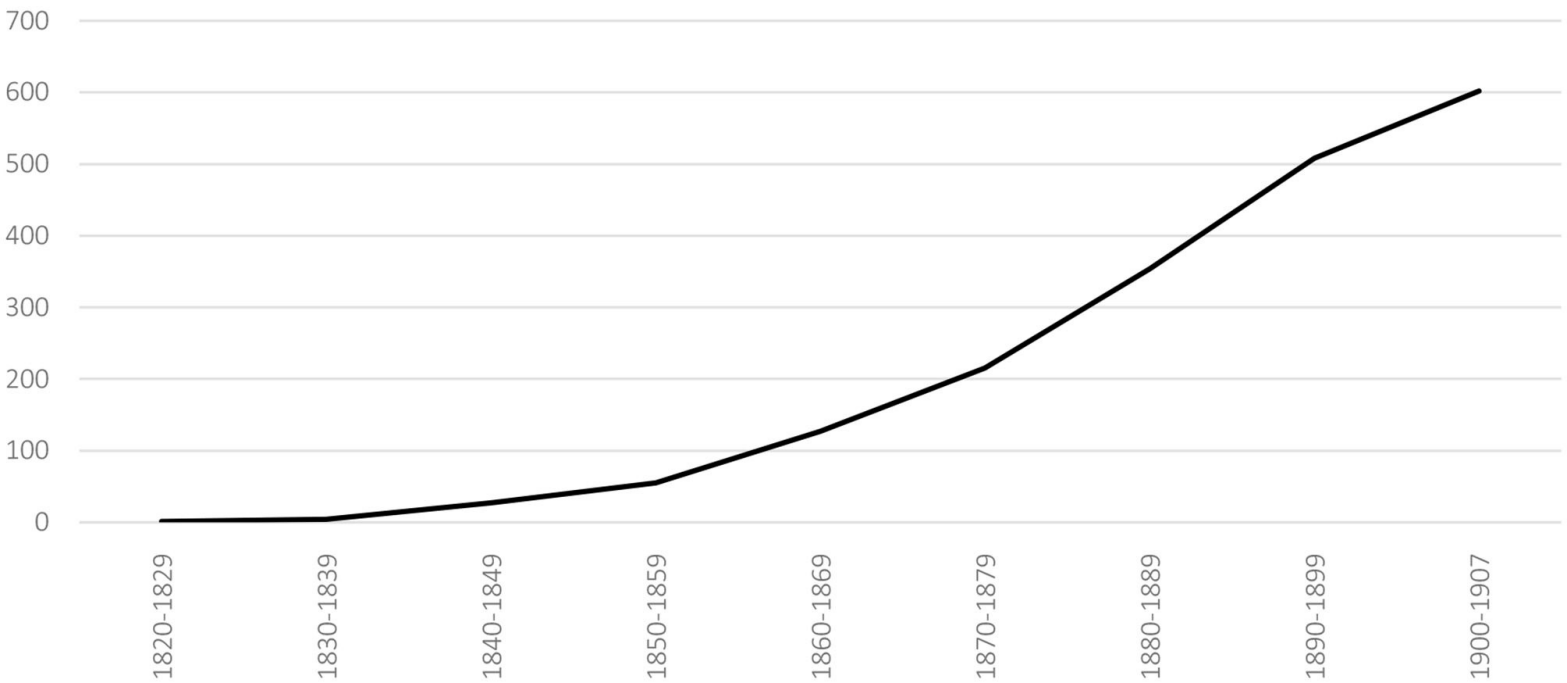
Number of local gymnastics clubs founded in Switzerland per decade (1820–1907) (SFG census of 1907).

Although the first gymnastics clubs were founded in Switzerland mainly by students in the first two decades of the nineteenth century (Czáka, 2008, p. 26), especially in the Bern-Basel-Zürich triangle, Figure 1 shows that the expansion of gymnastics clubs really began around the 1860's, before slowing down at the turn of the century.

About 450 clubs were founded between 1860 and 1900 (SFG census of 1907), while the SFG had a total of about 530 clubs in 1900 (Spühler et al., 1907, Annex 3). It is interesting to note that this trend was part of the wider development of associations in Switzerland in all fields. Indeed, Hans-Ulrich Jost estimates that there were about 30,000 associations and societies in Switzerland in 1900, half of which had been created after 1880 (Jost, 1991, p. 14, 15). In this context, the political role of the associations was growing in importance. The strengthening of the federal State, which had been under way since the 1870's (Meuwly, 2013), required, among other things, institutions that were capable of directly assisting it in certain governance tasks (Jost, 1991, p. 11), especially when successive governments chose to keep the size of the federal administration quite small and having the network of the associations function as a parallel one.

In this context, the SFG made an active contribution to the affirmation of the Swiss nation after 1848 and gradually assumed a “parastatal” function in the physical training of a specific section of the youth, so that in some of the speeches given by the gymnasts the SFG is mentioned in the same breath as the federal State (Jost, 1986). For instance, in 1907 the authors of the *Festschrift zum 75 jährigen Jubiläum des Eidg. Turnvereins* [SFG] *(1832–1907*) (Spühler et al., 1907) described the association as a “gymnastics state” and made a direct comparison between the evolution of Swiss State structures and that of the SFG's mode of governance:

“Whoever compares the present institutions of our gymnastics state, which […] are a reflection of our republican state institutions, with those of earlier and earliest times, will find the latter immensely primitive. They were; but the conditions to which our first constitution [that of the new state of 1848] was adapted were also small.” (Spühler et al., 1907, p. 20)

Beyond the patriotic symbol and the will to assert the national importance of the gymnastics institution which would function as a “miniature State,” a parallel can indeed be drawn between the evolution of the SFG's institutional structures and those of nineteenth -century Swiss politics, where actors tried continuously to balance the powers between the federal, the cantonal and the local levels. During the first 40 years of the SFG, the management of the organization was closely linked to the annual federal festivals. Between 1832 and 1869, the organizing club each year was awarded the central governance of the SFG and organized the general assembly during the festival. The system took advantage of the theoretical presence of members from all over the country during the festivities to hold votes, following the traditional model of direct democracy: the “landsgemeinde” (Spühler et al., 1907, p. 26). This model of “rotating presidency” can be compared to that of the *Vorort*, or *Canton directeur*, which between 1815 and 1847 saw the cantons of Bern, Zurich, and Lucerne handing over the federal destiny every 2 years, in an Assembly called the “Diet” which brought together delegates from all the confederated cantons before the creation of the federal State of 1848.

However, this itinerant mode of governance of the SFG was called into question from the early 1860's, precisely when the significant development of gymnastics clubs began and the practice became truly national in scope. Indeed, the general assembly did not sufficiently represent all the Swiss gymnasts, whose massive participation in the federal festivals remains complicated (Spühler et al., 1907, p. 26). Facing the complexity of centralization at a national scale, when the national and unified railways were still only projects, cantonal gymnastics societies (which bring together all the clubs of a single canton) were created from the 1850's onwards, a dynamic which initially generated tensions with the hegemonic will of the SFG (Spühler et al., 1907, p. 30). To counter this problem, a change in SFG statutes in 1861 set up a representative assembly of the delegates. Following the idea of the semi-direct Swiss democracy in which people and cantons are represented by members of parliament, each local club was now represented by delegates (one delegate for every 30 members). The function of this assembly was mainly legislative. It aimed to “promulgate or amend constitutional decrees and regulations [.]” (SFG, Statutes, Soleure : 1861, p. 5) and thus goes hand in hand with the executive function of the central committee.

**Figure 2:**
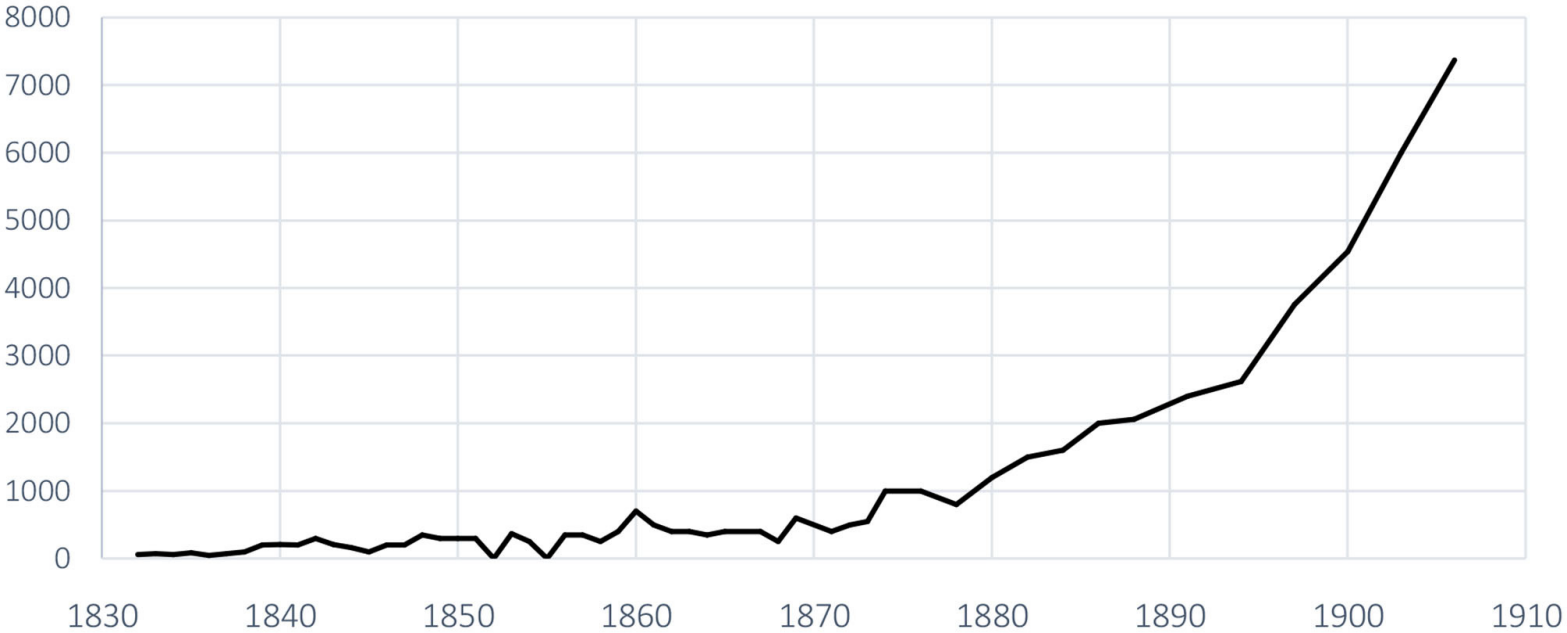
Evolution of the number of participants in the Federal Gymnastics Festivals by iteration (1832–1906) (Triet and Schildknecht, 2002).

Looking at Figure 2, the number of participants in the federal gymnastics festivals only began to increase steadily from 1880 onwards, with the milestone of 1,000 participants being passed in Lausanne in 1880, and then rose to 7,000 in 1906. To illustrate the fact that the federal festivals do not by any means gather together all the gymnasts in the country, the particularly well-attended—for the time—festival of 1860 in Basel, a dynamic canton in the beginnings of Swiss gymnastics, with 700 participants, barely managed to bring together half of the members of the SFG (Spühler et al., 1907, Annex 3). It should be noted that the attendance declined again during the decade 1860–1869.

The beginning of the 1870's marked the official break between the management of the SFG and the organization of federal festivals. Indeed, in late 1869, it was decided to create a permanent central committee whose members served for two years (Niggeler, 1882, p. 121). This new permanent central committee was composed of six members (including a president) elected by the members of the SFG from the 12 candidates proposed by the assembly of delegates. Only one part of the old system persisted: a seventh member was elected annually by the club that organized the federal festival (SFG, Statutes, Bâle : 1873, p. 7).

This break, which saw a drastic reduction in the importance of the organization of federal festivals in the governance of the SFG, must be put into context. While the new and more centralizing federal constitution was rejected in 1872 and accepted in 1874 (Meuwly, 2013), the 1870's saw the organization of the last truly political federal festivals, in their initial dimension as forums for the liberal-radical current. The official political parties gradually assumed this function, while the federal festivals became more and more independent from politics and more specifically focused on their own discipline (Schader, 1992, p. 812). With this in mind, a “technical commission” was founded within the SFG in 1871 alongside the central committee. Composed of three members, of whom at least one came from the central committee, it aimed to “pre-advise or decide purely on questions of gymnastics techniques” (SFG, Statutes, Bâle : 1873, p. 9).

While the increase in the number of participants in federal festivals reflected the expansion of gymnastics in Switzerland during the second half of the nineteenth century (see Figure 2), it should also be noted that, initially organized every year, the festivals took place every 2 years from 1874 onwards precisely to encourage participation, even though at the time a trip inside the country was very costly and the gymnasts had little or almost no free time. For instance, the Factories Act of 1877—which “limits” work to 65 h a week including Saturdays and prohibits child labor—was then genuine social progress. In the same vein, the festivals were then held every 3 years from 1888 onwards.

In addition to this organizational change, it should also be noted that the democratization of the railways can also explain the increased participation in festivals (Fritzsche et al., 2001, p. 85). As mentioned in the report of the organizing committee of the 1900 Festivals in La Chaux-de-Fonds, while the Federal Railways Company, founded in 1898, seemed reluctant to grant preferential prices for gymnasts, the committee nevertheless obtained free return tickets from local companies during the period of the festivals, and special trains were chartered from Zürich (SFG, La Chaux-de-Fonds, 1900, p. 69–70).

To continue with the turning point of the 1870's, the acceptance of the new federal constitution of 1874 saw the SFG's direct involvement in physical education gradually becoming institutionalized. Indeed, in 1866, the overwhelming Prussian victory at the Battle of Sadowa prompted Emile Welti, the Federal Councilor in charge of the military department, to campaign for a more centralized army and better training for military service (Burgener, 1952, p. 81). Under the new constitution, which, among other things, strengthened the centralized power of the federal State in military matters (Meuwly, 2013; Jaun, 2019), “gymnastics […] is recognized [by political bodies] as being of national and military utility” (Burgener, 1952, p. 98). Physical education thus became compulsory for Swiss boys over 10 years old until the end of primary school—back then, secondary or high school did not exist in Switzerland—and became the only discipline driven by the federal State, whereas the rest of public schooling remained in the hands of the cantons (Bussard, 2007, p. 26).

The extra-parliamentary *Commission fédérale de gymnastique* (CFG) was also created in 1874, having it first meetings in the very 1st months of 1875, in order to set up the new rules and to define the content of a federal textbook for gymnastics at school. The CFG was initially composed of four members, three from the school environment and one, a senior officer, representing the federal military department (Eichenberger, 2001, p. 85). The initial composition of the CFG included representatives from the SFG's central committee, whose stabilization since 1869 favored long-term project management and partnerships with the State (Jost, 1986). Thus, the founding members of the CFG include Johannes Niggeler, SFG president since 1870 and disciple of Adolf Spiess (Horlacher, 2017), who also served as a physical education teacher, and Carl-August Rudolf, another member of the SFG central committee from 1870 to 1873 (Flatt, 1945), along with Wilhelm Schoch and Johann Jakob Egg, two of the pioneers of the *Société Suisse des Maîtres de Gymnastique* (SSMG), created in 1859 in order to coordinate early attempts to introduce gymnastics into schools (Müller, 1910).

On the financial side, the central fund of the SFG was mainly provided by the half-yearly contributions of members of each affiliated club (50 cents per gymnast in 1873), the interest on the association's capital and a 10% share of the profit of each federal festival (SFG, Statutes, Bâle : 1873, p. 11). However, while compulsory physical education was about to be introduced, the SFG received federal subsidies from 1873 onwards, to organize teacher training courses (CHF 1000.- in 1873, representing more than a quarter of the association's annual income) (Spühler et al., 1907, Annex 7). Indeed, the SFG took partial charge of the federal courses of physical education for teachers from the beginning of the 1870's, but then as an official duty from 1889 until 1911 (Burgener, 1952, p. 155). This process led to the SFG being legally recognized by the State in 1907, following the adoption of new military legislation (Burgener, 1952, p. 187), as officially responsible for the training of young men at the end of compulsory schooling within the framework of what is known as “preparatory instruction” (Eichenberger, 2001, p. 88).

### Gymnastics and Statistics

Bearing in mind that federal associations represented first a “proto national cohesion” (Im Hof and Bernard, 1983, p. 10) before 1848 and with the idea, proclaimed loud and clear by the leaders of the SFG, that they later took on the function of “miniature States,” the SFG conducted and published statistical surveys from the 1850's onwards. The emergence of statistical surveys within the association suggests that it was fairly stable in its structure and had enough members in the mid- nineteenth century to undertake such census work. These statistical surveys were institutionalized within the SFG in 1852 through the new article 15 of its statutes which charges the central committee to “monitor” the development of the organization (SFG, Statutes, Genève : 1852, p. 10). Finally, in 1861 new statutes explicitly mention that the central committee must publish statistical records of gymnastics clubs and that all clubs wishing to be affiliated must provide a statement of the number and age of their members (SFG, Statutes, Soleure : 1861, p. 8–11).

The purpose of those censuses was to measure the growth of the SFG, in the manner of national statistical offices founded for the purpose of building, unifying and administering young nation-states during the first half of the nineteenth century (Desrosière, 1993, 16–17). This interest of the SFG in the statistical statements within its clubs underlines once again the extent to which it reflected the federal State and the “parastatal” function of the institution, which quickly played a role in the census of the young Swiss nation, whereas the first inventory of the population of the federal State dated back to 1850 (Humair, 2004, p. 804) and the Federal Statistical Office was only created in 1860 (Jost, 1995). However, it is important to stress that those dynamics were not specific to gymnastics; just a few examples of a broader landscape include the installation of a permanent committee at the head of the Swiss Shooting Association, in 1877, which was directly followed by several statistical projects (Gamma, 1924) and the founding of a peasants union (the *Union Suisse des Paysans*) in 1897, which had as its main objective the establishment of agrarian statistics (Humair, 2004, p. 646).

In its official report of the year 1853–1854, the SFG had already published a table showing the gymnastics clubs that had responded to its request, with the number of members, their year of affiliation to the SFG and the names of the individuals making up their central board (SFG, Annual Reports, 1853–1854, p. 3–4). The early date of this gymnastics survey—which takes precedence over the state institutionalization of statistics—supports the fact that until the beginning of the 1860's, statistics in Switzerland were mainly collected by notables (pastors, doctors and magistrates) and by public utility societies (Busset and Le Dinh, 2001, p. 58), in which the SFG can be included.

In order to work on gymnastics statistics of the time, the main empirical resources of this paper are three surveys carried out by the SFG during the second part of the nineteenth and early twentieth centuries. The oldest one, carried out in 1873, shows, according to the leaders of the SFG, “a constant increase in the number of gymnastics clubs, which corresponds to the uninterrupted diffusion of gymnastics in Switzerland” (Reports on the SFG for 1873, Bern, 1874, p. 4). The fact that this survey took place in the same year as the first federal grant for the SFG and 1 year before the introduction of compulsory physical education is not insignificant and shows that statistics were also an instrument of power for the SFG. Indeed, the figures had to show to the political leaders the reach of the SFG and its clubs, in particular in the field of youth, in order to consolidate the partnership of the gymnasts with the State. Thus, it should be noted that, at the beginning of 1874, the federal Department of the Interior, in order to deliver for a second consecutive year the grant of CHF 1000.-, asked SFG's central committee to send, in addition to its budget, the official report of the SFG's activities in 1873 (Reports on the SFG for 1873, Bern, 1874). This contains the final version of the 1873 statistical survey (SFG, Central Committee, January 17 and 18th 1874).

The most recent survey, conducted in 1907 as part of the 75th anniversary of the SFG, also aimed to establish a broad statistical statement of the members of the institution. The goal was to trace its historical development in the context of the writing of the commemorative book *Festschrift zum 75 jährigen Jubiläum des Eidg. Turnvereins (1832–1907*). However, it should be noted that this census also came at a key legislative moment, with the adoption of the new military legislation of 1907, which fully institutionalized the parastatal role of the SFG, as previously discussed, along with the introduction of mandatory rifle practice creating the same role for the *Société Suisse des Carabiniers* (Gamma, 1924).

Following the traditional federalist scale of the political system, tables were sent in both cases (1873 and 1907) to the cantonal gymnastics associations in which they were to fill in, among other things, the names of all the local clubs in the canton, the date of their foundation, the number of active members and their ages (under 20 years old/between 20 and 30 years old/over 30 years old for 1873; under 20 and over 20 years old for 1907). The cantonal associations had then to return the completed tables to the SFG. This interest of gymnastics leaders, at the turn of the nineteenth and twentieth centuries, in statistics and figures, as a historical and governance tool, is also to be seen as part of the administrative revolution that had been at work since the 1890's, which saw the deployment of new forms of large-scale processing of written and numerical data as well as a wider circulation of these data (Gardey, 2008, p. 16). Furthermore, the SFG used a private professional service to process the information it collected on its member clubs in 1907, entrusting the Steiner statistical office in Bern with this task (SFG, Central Committee, December 7th−8th 1907).

## Getting to Know Youth, A Way of Attesting the National Utility of Gymnastics in Switzerland

Besides the fact that the categories changed between the 1873 and 1907 surveys, placing more importance in 1907 on the turning point of reaching the age of 20, one must also emphasize that youth had been at the core of the SFG's nation-building role since the middle of the nineteenth century, as a tool to defend the “homeland against alien hands and to learn about patriotism” (Le Gymnaste, 1860, p. 10).

### The SFG at the 1896 National Exhibition: Between Power Display and Physiological Measurement of Youth

In the framework of the second National Exhibition organized in Geneva in 1896, statistics supported by graphs and maps were also a means of emphasizing the SFG's contributions to nation building, especially by illustrating the benefits of gymnastics on the bodies of Swiss youth. The National Exhibition was intended to:

“provide an overall picture of Switzerland's productive capacity in the fields of science, industry, arts and crafts, fine arts, agriculture, public education and the social economy. It should make the Swiss people appreciate its own strengths, open up new domestic markets for national production and give them a concrete sense of the importance of its activities.” (Official Guide to the 2nd Swiss National Exhibition, Rey-Malavallon : Genève, 1896, p. 63)

It was in order to be part of this idea of the perception of “national strengths” that the Central Committee of the SFG suggested taking part in the Exhibition at the 1894 General Assembly held in Solothurn. The proposition was unanimously accepted a year later after the presentation of a more concrete project (Spühler et al., 1907, p. 64). As evidenced by the words of President Erwin Zschokke during a working meeting of the Central Committee in September 1894 (SFG, Central Committee, September 22nd−23rd 1894), the SFG—which was to have an exhibition area of 16 m^2^ in pavilion number 21, where several social and professional associations would be brought together—would have to present to the public, among other things, a topography of gymnastics in Switzerland (“Turn. Topographie der Schweiz”), a graphic representation of the growth of gymnastics clubs and information on the members (age, state of health) to demonstrate “the influences of the practice of gymnastics.”

In the discussion around the project, Ernest Baud from Geneva, who was also the president of the office of pavilion number 21 (Gavard, 1896, p. 70), insisted that illustrating the health benefits of gymnastics would be the most valuable aspect of the SFG's participation in the exhibition. He added that such scientific work based on measurements and observations should be provided by a doctor or a professor. Finally, as subjects for the study, it would be a matter of selecting young people (“Jünglinge”) who “cultivate” gymnastics, in order to compare them with those who don't practice it (SFG, Central Committee, September 22nd−23rd 1894).

Thus, our third source survey is the result of the SFG's participation in the National Exhibition and of the project that SFG leaders had been working on since September 1894. The survey was carried out during the year 1895 in order to provide body measurements of individuals members of local clubs that were affiliated to the SFG (chest circumference, arm circumference and general state of health of gymnasts). This survey was similar to the administrative procedure organized in 1873 and 1907, but was conducted by doctors as requested by Ernest Baud. Participants were to give their date of birth (which tells us exactly how old they were rather than only a range) but also how long they had been practicing gymnastics, which is particularly interesting in the context of this paper.

From our three source surveys, three data tables can be established. The first two (1873 and 1907) bring together local gymnastics clubs (89 clubs for 1873, 651 clubs for 1907) affiliated with the SFG, their date of foundation, the canton they belong to, the number of active members and the age brackets of these. The third data table is based on the 1895 survey which unfortunately is not available in its entirety in the archives. It provides the age in 1895 and the age of starting gymnastics practice for 914 individuals, who account for ~9% of all the active members of the SFG in 1895, distributed across 36 clubs (mainly from the Swiss German part of the country).

Regarding the body measurements, it is not known whether the SFG followed the will of Ernest Baud and compared young gymnasts with non-practitioners. However, the anniversary book of 1907 indicates that results, based on three successive measurements taken on only 329 gymnasts, “demonstrated in figures the effects of gymnastics on the muscles and chest circumference, a work which has received full attention in scientific circles” (Spühler et al., 1907, p. 65). The short phase of taking measurements (only 20 months between the first discussions around the survey and the beginning of the National Exhibition) and the selection of 329 individuals from a sample of more than 914 lead us to be cautious about the real scientific validity of these results. We can also assume that the leaders of the SFG had selected the data to present their results, selecting “young” gymnasts only. Indeed, the sample of 914 gymnasts includes entire clubs and also contains individuals over 25 years old, who don't correspond to the definition of “youth” within the SFG, a definition that we must now focus on by describing pragmatically the trends of club membership in terms of age and seniority.

### The Definition of Youth in the SFG

While the very notion of “youth” spread throughout the nineteenth century, its predominant place in the discourses and activities of the SFG, particularly in the areas of national unification and defense, reflects the magnitude of the political function that was quickly attributed to it (Bantigny and Jablonka, 2009, p. 11). However, by examining the age of the SFG members, it is now a question of studying the real amplitude of this “youth” in the national development of Swiss gymnastics during the second half of the nineteenth century. Besides, the aim is also to determine in fact what was meant at that time by a “young” or an “old gymnast” in Switzerland.

First of all, it must be specified that the SFG, in its definition of youth, left out a whole section of it for a long time by setting the minimum age of its members at 16 (Bussard, 2007, p. 151–158). Compulsory physical education since 1874 and the process of establishment of public schooling (in particular the compulsory and free primary school) can explain the position of the SFG and its choice to consider only boys over 16. It may not have considered it necessary to integrate these age groups because in theory they already practiced physical education at school, partly under the aegis of the SFG which took care of a growing part of the courses for teachers (Flatt, 1915).

Of the three censuses studied, only the one of 1895 gives the exact age of the gymnasts. The average age of the 914 subjects it contains is 19.8 years old. It is important to note that the average age of gymnasts is a relative indicator of youth at a time when the average life expectancy at birth for Swiss men was 45.5 years due to the high infant mortality rate. Above all, it should be noted that reaching the age of 20—which not everyone did—meant having already lived about one third of one's life. Indeed, a 20-years-old Swiss man could expect to live up to 60 years on average during the decade 1890–1900 (HSS, Average life expectancy, by sex, from 1876 to 1995).

We note that the trend among the subjects of the study was not to join the SFG at the minimum allowable age of 16. Indeed, gymnasts only joined a gymnastics club at an average age of 17.8 years. The indicator of the length of the gymnastic career is particularly interesting because the sample shows that the population is very young in terms of years of practice. On average, in 1895 the subjects had only 2.2 years of activity in their club. On the other hand, this very low seniority of members is not reflected in the structures, which would logically see recently founded clubs principally attracting young and novice members.

Indeed, the seniority of the clubs from which the gymnasts of the 1895 census come is 27.5 years on average. Similarly, the more long-established clubs did not have an older membership, making it quite obvious that a high turnover might be the rule, the SFG's clubs mostly providing space and time for youth between school and recruitment in the army, as set out in the federal military organization of 1874. For instance, in 1895 at the “Zurich Alten Sektion,” the third oldest club in Switzerland founded in 1820, the average age of members was 20.3 years and the average duration of membership was 2 years.

**Figure 3:**
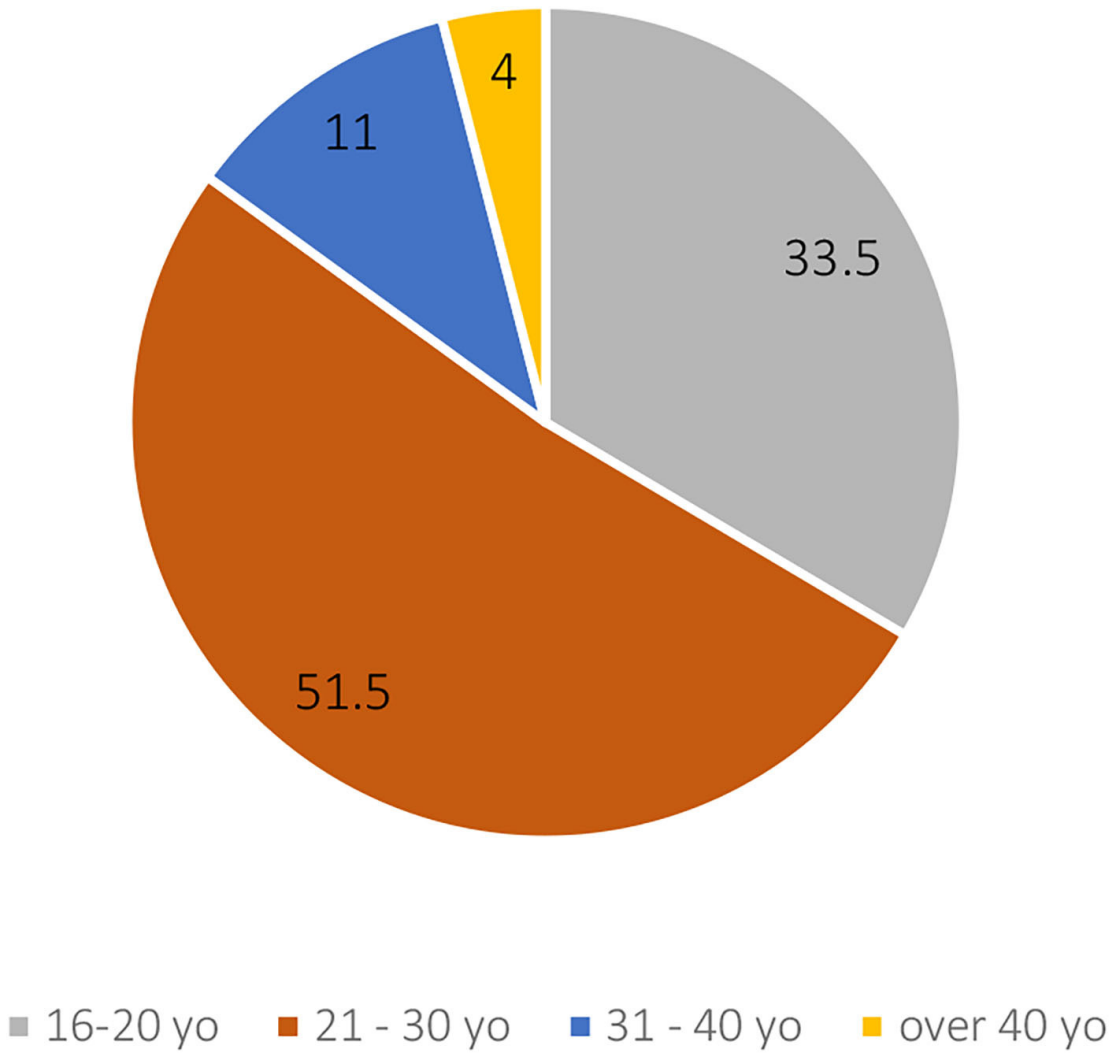
Percentage of age groups of the membership of 89 SFG clubs in 1873 (SFG census of 1873).

The censuses of 1873 and 1907 classify the gymnasts by age group, rather than the precise age of every member, which allows us to discern some trends in the SFG population. The 1873 survey is the most precise and offers four distinct ranges, represented in Figure 3, showing a clear predominance of gymnasts under 30 years old. It should be noted that the 16–20 age group still represented 33% of the members although that age group is half the age range of the others (5 years instead of 10).

The commentary of this census—which officially appeared in the 1874 annual report of the SFG distributed to all affiliated clubs—explicitly links the 16–25 age group to an understanding of what “youth” is, as opposed to a “more mature age:”

“From the table we've just drawn up, we can see that our clubs aren't mainly recruited from the more mature age groups, […], but that they are mainly populated by the younger generation. It would have been even more striking if the period from the 20th to the 30th year had been divided into two parts.” (Reports on the SFG for 1873, Bern, 1874, p. 4)

According to this quotation, the majority of the gymnasts in the 21–30 age group would be concentrated between 21 and 25 years old. Moreover, the insistence on this division and the fact that the 30 and 40-year-olds are already defined as belonging to the “mature age” group is characteristic of a time when being 30 years old meant, on average, being beyond the middle of one's life (HSS, Average life expectancy, by sex, from 1876 to 1995). This life expectancy that lowers the “boundaries” of old age may partly explain the drastic drop in the number of people practicing once they pass the age of 30.

**Figure 4:**
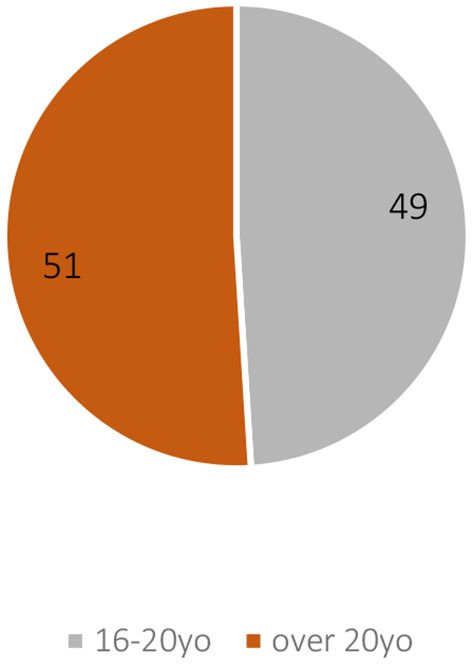
Percentage of age groups of the membership of 651 SFG clubs in 1907 (SFG 1907 census).

The 1907 census is much larger (651 clubs out of a total of 690 in 1907) but unfortunately less accurate than the 1873 survey in terms of age, presenting only two categories of classification. Figure 4 nevertheless retains the 16–20 age group, which alone in 1907 accounted for 49% of the SFG's membership, compared with 33% of the 1873 sample. This upward trend reflects the fact that the “younger generation,” and in particular the under 20's, became even more important over three decades within the total population of gymnasts. This pivotal period between 1880 and 1900, which experienced a particular boom in gymnastics in Switzerland, thus saw the SFG's membership become even younger, while being responsible for the nationalization of gymnastics during the post-1880 wave already described and which affected not only gymnastics (Jost, 1991).

It is now necessary to look in more detail at the examples of Zurich—the third canton to present the most gymnasts over 20 years old—and of Berne—the fifth canton to present the most gymnasts over 20 years old. In these particular cases, this is partly because the two cantons are pioneers in the foundation of “Männerturnvereine” or “Sociétés d'homme” that designate clubs for “senior gymnasts.” In 1907, the membership of these clubs represented 230 of the 1,703 individuals over 20 years old in Zurich (distributed among four clubs) and 318 of the 1210 individuals over 20 years old in Berne (distributed among three clubs), the canton where the first “Männerturnverein” was founded in 1846 (SFG 1907 census).

These clubs for “senior gymnasts” are particularly interesting in the context of this article, which paradoxically aims to examine youth in Swiss gymnastics. Indeed, the great majority of Swiss gymnasts were under 25 years old during the second half of the nineteenth century and the SFG did not seem to have any problems targeting what it defined as “youth,” an objective which was, moreover, totally assumed in its official aims. However, the greatest difficulty for the SFG seems to have been to sustain the commitment of members within the clubs, members who, as we have seen, generally had a very short gymnastics career at this critical period that follows the compulsory school years. The comment made by the SFG on the 1873 census highlighted the problem and enjoined the young gymnasts to act to keep the older ones involved in the clubs, and refrain from using representations that associate old age and physical decline. In addition, the “Männerturnvereine” were directly designated as a good way to prolong the commitment of the gymnasts:

“It is deplorable to ‘keep out’ the most mature elements [gymnasts] [.]. Young people must try to hold on to the old with all their might, but they must also take care of them and not directly turn away from what they see as less solid and less attractive. Wouldn't it be possible to set up an organization [clubs for senior gymnasts] in all big cities, like the one in Bern?” (Reports on the SFG for 1873, Bern, 1874, p. 4–5.)

An examination of the “Männerturnverein” membership of the city of Zurich through the 1895 census tells us more about those who were considered “old gymnasts” in Switzerland at the end of the nineteenth century. The oldest gymnast in the clubs was an honorable 47 years old followed by a 43-year-old and two 40-year-old gymnasts. In fact, these subjects could claim to live to the age of 65.5 on average, but they were already exceptional cases in terms of their longevity, which was coupled with a commitment to a gymnastics club. However, 62% of the members were under 30 and the average age was 26.3 years old, which is only 8 years older than the average age of standard clubs (SFG, Census 1895 SFG census).

The case of these clubs for “senior gymnasts” with a surprisingly young membership underlines once again the need to question our traditional boundaries in terms of youth and old age within the framework of this historical approach to gymnastics in Switzerland. With regard to the life expectancy, and while the Factories Act of 1877 mentioned earlier reflects the hardship of the weekly workload, the youth and the short career of gymnasts are probably characteristic of an era in which long-term investment in clubs dedicated to physical activity remained complicated. The nationalization and expansion of gymnastics in Switzerland was therefore mainly achieved through its youth. In fact, the spatial and temporal dimensions show that the increased development of gymnastics clubs in Switzerland between 1880 and 1900 mainly affected individuals under the age of 25, if not under the age of 20.

It is necessary to end this chapter by highlighting the eminently masculine character of the youth which made up the membership of the gymnastics clubs studied. At the level of physical education firstly, young women's bodies were not invested with the virile role of patriotism and national defense. Although initiatives and “specialized” manuals have existed since the end of the nineteenth century, it was not until 1972 that women's physical education in schools was formally made compulsory. The development of women's gymnastics in Switzerland is based “on very classical, even conservative, aesthetic and physical standards, around the maternal figure that women should aspire to remain” (Quin and Mayencourt, 2020, p. 1). The SFG has therefore long been a man's world that doesn't directly include women. Indeed, l'*Association Suisse de Gymnastique Féminine* (ASGF) was founded in 1908, with the status of a “sub-association” of the SFG. The historical study of the ASGF's national expansion and its relationship to youth has yet to be completed.

## A “Gymnastics State for Youth” Facing Competition

The SFG's unifying and patriotic rhetoric with regards to Swiss youth perhaps never resonated as much with the political context as it did at the time of the publication of the 1848 statutes, when the cantons were emerging from the *Sonderbund* civil war. In 1847, the conflict revealed a deep split between the industrialized areas, mainly governed by the liberal-radical political current, and the rural areas mainly run by conservative Catholics (Humair, 2009, p. 58–59). The victory of the first, which translated into the creation of the central state, did not mean that the tensions between the two camps had completely subsided. The liberal-radical hold on federal politics in the early years led the conservative Catholics to withdraw into a cultural and traditional “ghetto,” although they began to organize themselves politically countrywide around the 1860's (Altermatt, 1994, p. 71–73).

### The SFG's Expansion From Chiasso to Basel and From Geneva to St. Gallen

Beyond the process of development of a Federal State that Switzerland had been experimenting with since the early years of the nineteenth century, cantons (or regions) continued to be key players to understand the political, cultural, and social processes influencing the rise of gymnastics, creating the conditions of a heterogeneous landscape for a “nationalized cultural practice.”

Moreover, the resistance to the federal State that was present in some cantons echoes the desire for national unification in the discourse of gymnasts who designed their practice as having a real national scope and unifying potential. This is shown by SFG participation at the National Exhibition of 1896 which aimed to demonstrate the extent of gymnastics clubs throughout the country—even if in reality this was very uneven according to the cantons as we will see—as well as the physical benefits of the practice on the bodies of Swiss youth in the whole country.

**Figure 5:**
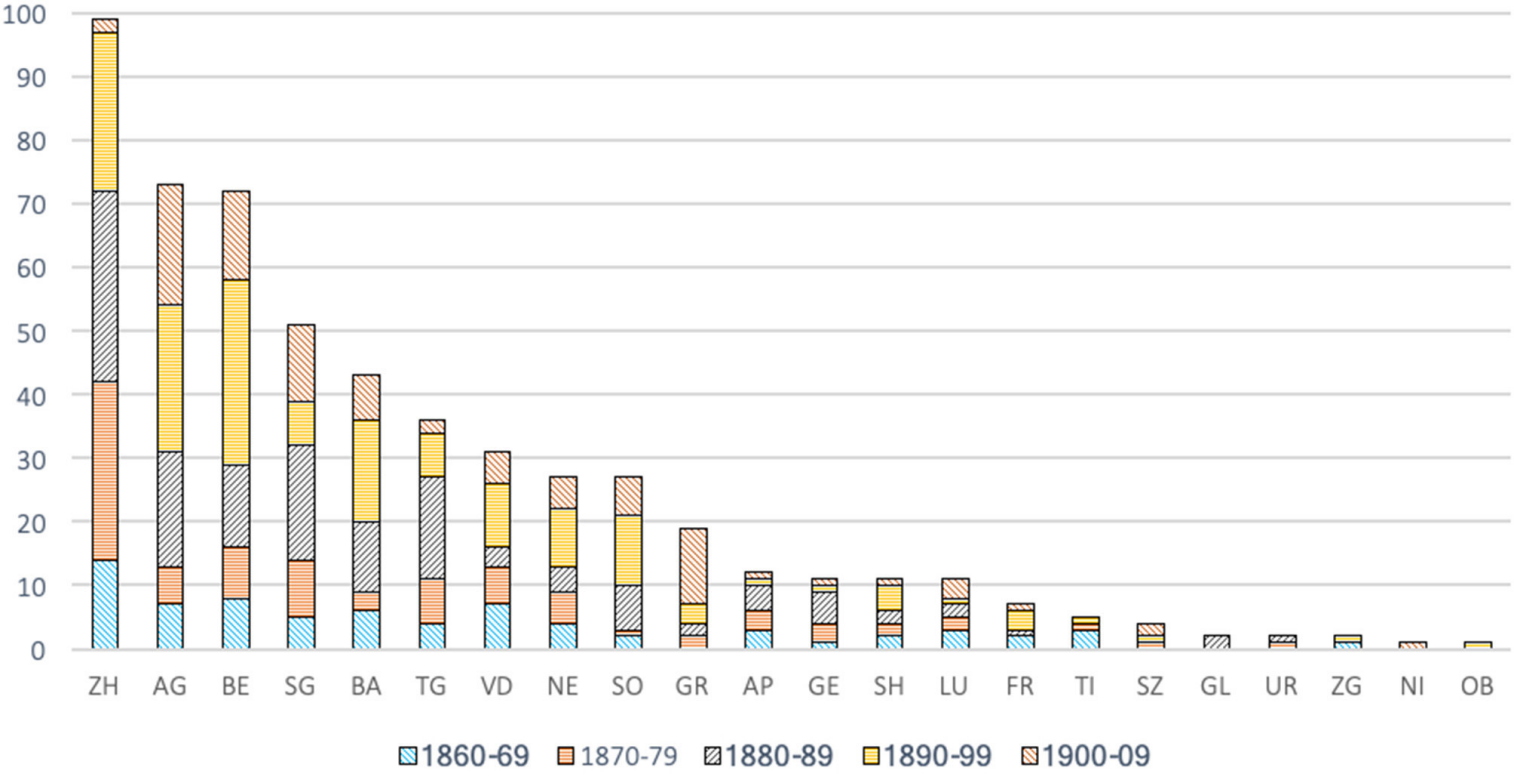
Number of gymnastics clubs founded by decade by canton (1860–1907) (SFG census of 1907).

Figure 5 shows the development over time of gymnastics clubs in Switzerland, by canton and by decade from 1860 until 1910. At first glance, this figure allows us to see at once that there has been at least one gymnastics club in each canton—besides Valais which was not part of the census, but which had eight clubs in 1895 (Spühler et al., 1907, Annex 3)—only since 1906 when the club in Stans in Nidwalden (NI) was founded.

**Figure 6:**
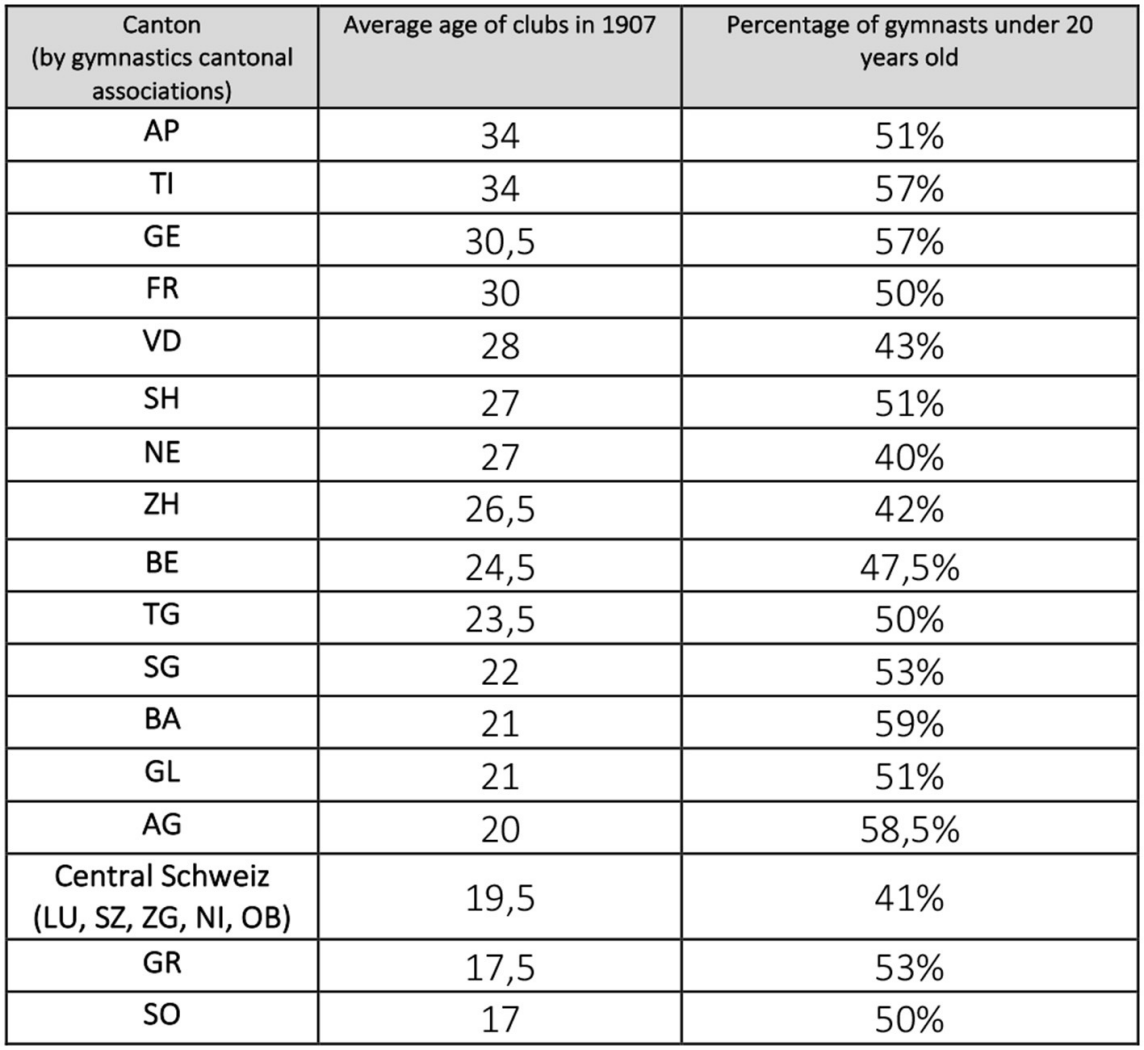
Comparison of the age of the clubs according to the proportion of gymnasts under 20 years old (SFG census of 1907).

The first stage of the expansion of gymnastics clubs during the decade 1860–1869 mainly concerned western and northern Switzerland, the two mainly industrialized and Protestant parts of the country (Humair, 2009). For 1860–1869, 51 of 68 clubs founded are in the cantons of Zurich (ZH), Bern (BE), Aargau (AG), St. Gallen (SG), Basel (BA), Vaud (VD), Thurgau (TG), and Neuchâtel (NE). We note the strong dynamism of the canton of Zurich from 1860 onwards, which was confirmed during the following three decades, so that, as shown by the few clubs founded in the whole canton of Zurich between 1900 and 1907 (only two), the process of institutionalization of gymnastics probably reached a first milestone around 1900. Following the increase of the population in the city of Zurich, it is also to be noted that the city itself had more gymnastics clubs than other towns in Switzerland: already 15 by 1879 and more than 20 just before 1900, as the city quadrupled its population between 1850 (≈50,000 inhabitants) and 1900 (≈200,000 inhabitants).

Besides the case of Zurich, which precedes the trend from the 1880's, the main peak of the creation of clubs in the west and north of the country was in the period from 1880 to 1900, especially along the northern border from Basel to St. Gallen, where the rise of the Swiss industrial complex happened first. Interestingly, it is also in the same regions (notably SG, BA, and ZH) (Koller, 2016) that new sports such as football would find their place and new players, beyond the dichotomy of “tradition–modernity” and ideological conflicts between gymnasts and followers of the new sports.

Keeping Figure 5 in mind, we cannot discern by looking at Figure 6 a trend that would see the cantons with a later development of gymnastics having younger memberships, in particular individuals <20 years old, and vice versa.

**Figure 7:**
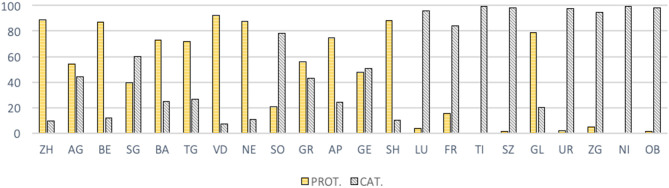
Proportion of Catholic and Protestant by canton in percentage (1880) (HSS, Federal population census of 1880).

In terms of religious confession, Aargau (AG) and St. Gallen (SG) are cantons where the difference in proportion between Catholic and Protestant is quite small, and Catholics were in the majority in SG at the end of the nineteenth century (see Figure 7). However, they are located in industrialized areas dominated by the radical-liberal current. The fact that these two cantons experienced a rapid increase of the number of their gymnastics clubs (affiliated to the SFG and not from Catholic confessional circles), especially from 1880 onward, suggests that the criteria of liberalism, industrialization and urbanization outweigh the religious component in the diffusion of gymnastics. It also calls for a more precise “geographical analysis,” based on the example of Zurich, where the urbanization might have played an important role, and where, probably, the ramification of a rural exodus from its hinterland to the city itself concentrated a younger population in towns.

The opposite cases of Solothurn (SO) and Glarus (GL) are also emblematic: SO, mostly Catholic but a western canton, saw the foundation of 11 gymnastics clubs (also affiliated to the SFG and not from Catholic confessional circles), on its territory from 1890 onwards. The very “liberal” character of SO, despite its Catholic faith, must be emphasized here. For example, the canton did not take part in the conservative alliance of the *Sonderbund* which led to the civil war of 1847 (Humair, 2009, p. 66). Conversely, in GL, a rural canton close to central Switzerland but in which nevertheless 79% of people were Protestants in 1880, the development of gymnastics was almost zero, even considering the small population of the canton.

It is also important, therefore, to be aware that the “cantonal level” is probably not the best one from which to understand precisely the spread and flourishing of gymnastics. Aargau is a very good example, as shown in Figure 9 below, its northern districts (or municipalities) being more Catholic and showing a lower concentration of gymnastics clubs, and the same for the south of the canton along the Reuss river, at the border with the Catholic cantons of Luzern and Zug.

The alpine and rural canton of Graubünden (GR) is another good example, having a small difference in the Protestant–Catholic ratio similar to AG and SG, experiencing some development of gymnastics from 1900 onwards, but being also quite divided between valleys. We must also mention Ticino (TI) which shows a certain dynamism in the decade 1860–1870 with four clubs affiliated to the SFG founded in its main towns (Bellinzone, Lugano, Locarno, and Chiasso). TI is close in its characteristics to the rural, Catholic cantons of central Switzerland where gymnastics developed only timidly from 1870 onwards, and even later, from 1890 and 1900 on for Obwalden (OB) and Nidwalden (NI), independently of the confessional orientation of the gymnastics clubs and their institutional affiliation.

However, the Italian-speaking canton wanted to show its attachment to the confederation quickly through gymnastics, in particular thanks to the investment of radical political personalities in the movement (Marcacci, 2019, p. 141) such as Giovanni Jauch, a fervent defender of radicalism, deeply anti-clerical and president of the federal gymnastics festival organized in 1868 in Bellinzone. Like Solothurn, Ticino also did not take part in the *Sonderbund* alliance despite its Catholic faith (Altermatt, 1994).

Finally, it should be noted that the confessional Catholic gymnastics clubs are not included in our data, as they were not included in the SFG surveys. Thus, following the proclamation of the encyclical “Rerum Novarum” of 1891, they are part of an associative movement launched by the Catholic Church in order to update itself through modern means (Altermatt, 1994, p. 44). However, the extent of their development, which therefore began around 1900, was not comparable to that of the traditional clubs affiliated to the SFG. Besides, Urs Altermatt explains that Catholic associations (of all types) have to be considered as means of resistance against Protestant power, as demonstrated by their origination in cantons where political Catholicism had a smaller scope. Conversely, their development was weak in the cantons where political Catholicism was fully established (80).

**Figure 8:**
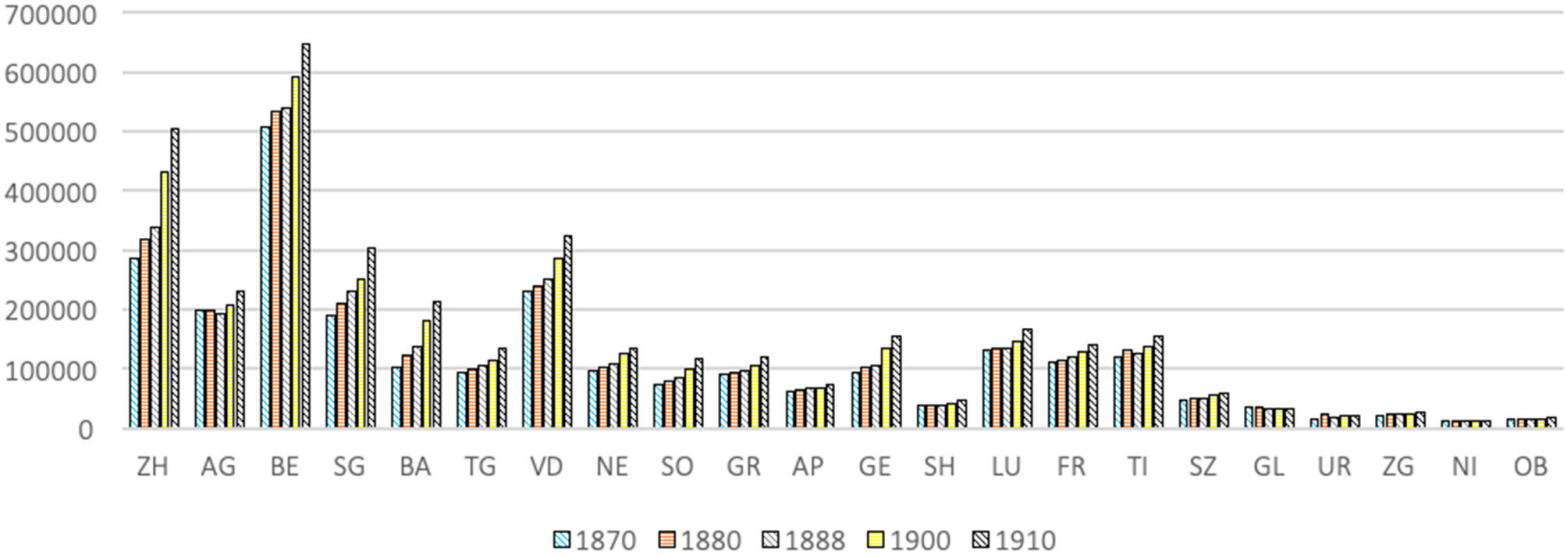
Population by canton (1860, 1870, 1880, 1888, 1900, 1910) (HSS, Federal population census of 1870, 1880, 1900, 1910).

In addition to the religious issue, Figure 8 shows that the population of the cantons must also be considered along with the spatial development of gymnastics in Switzerland. On the one hand, the cantons of Central Switzerland are less populated and, beyond the resistance to gymnastics practice due to their Catholic and rural nature, one should also be aware that they have a smaller pool of potential gymnasts. On the other hand, more populated cantons which are strongholds of the conservative Catholics, such as Luzern (LU) and Freiburg (FR), show a much more modest development of gymnastics than certain industrialized cantons in western Switzerland, which are less populated. It can also be noted that Aargau, which over the period from 1870 to 1910 had less than half the population of the canton of Bern, experienced a boom in the expansion of its gymnastics clubs from 1880 onwards.

It is a complex matter associating certain demographic shifts with a change in the founding of clubs. For Zurich, for instance, the sharp increase in population during the period from 1888 to 1900 does not correlate to an increase in the number of clubs—which had already increased—but, as the increase is almost only due to the population of the city of Zurich, it shows how vital it will be in the future to understand in more depth those underlying socio-demographic processes. For Bern, on the other hand, the demographic leap in 1900 could be associated with the fact that the decade 1890–1900 also saw the highest number of clubs founded in the canton for the whole of the period from 1860 to 1907. Furthermore, the question of population again highlights the predominance of industrialization as a factor in the development of gymnastics. Indeed, large cantons such as Vaud (VD), which were less industrialized, experienced a slower growth in their clubs than other, less populated but more industrialized cantons (such as AG, SG, BA, and TG), also being at the core of the development of modern sports such as football (Berthoud et al., 2016).

**Figure 9:**
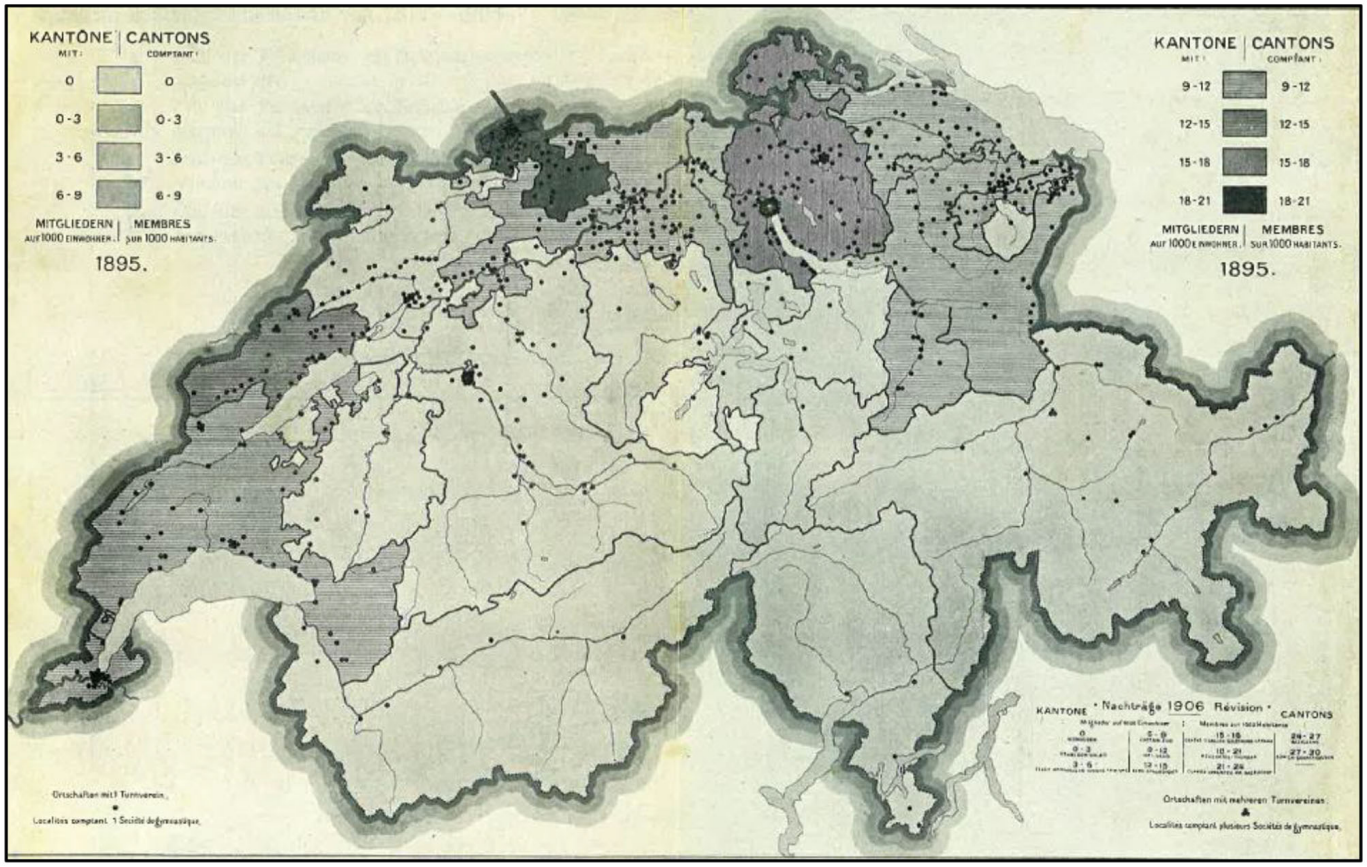
1895 map of gymnastics clubs in Switzerland (Spühler et al., 1907, Annex 3).

The map showing the “topography of gymnastics in Switzerland” (Figure 9) offers a good summary. The idea of this map, on which black dots represent all the gymnastics clubs in Switzerland, was launched by SFG president Erwin Zschokke, using data produced for the 1895 census. This map, whose physical format was imposing (Spühler et al., 1907, p. 1), was officially presented at the 1896 National Exhibition. It was intended to support the SFG's discourse to promote the national and patriotic dimension of gymnastics. The version shown here was published in the 1907 SFG anniversary book, updated from 1895 by the 1907 census. Interestingly, and to make the connection with the “mandatory gymnastics” introduced in 1874, the CFG received at its meeting of July 1907, a statistical report on gymnastics courses provided in each canton and, not surprisingly, the map is almost the same as that presented in Geneva in 1896. Basel offered all the apparatus and even an indoor infrastructure in each school, while schools in Obwalden, Fribourg or Ticino had to struggle; Obwalden for example apparently had no indoor facilities in 1907 (Burgener, 1952, p. 130).

The map highlights, above all, the arc containing the industrialized and mainly Protestant northern and western areas of the country as a particularly fertile ground for the development of gymnastics. It also emphasizes the urban component of the practice with important concentrations of clubs around the “biggest” cities such as Bern, Basel, Zürich, Winterthur, and St. Gallen. The presence of clubs in Catholic and especially rural areas is much more scattered. It is generally limited to one club per city and some villages, all located in the main valleys. One should also stress that while gymnastics was not homogeneously spread through the country, its growth was clearly following the roots of industrialization, especially those from a first industrial revolution based on control of the rivers, for energy production, but still leaving space for the expression of genuine “Swiss traditions” especially during the federal festivals of all kinds (Schader and Leimgruber, 1993).

### From Nationalization to New Competitions

The new regulation of the federal gymnastics festivals adopted by SFG in 1854, which saw the addition to competition programs of typically Swiss folkloric practices such as wrestling, stone throwing and flag handling, marks the official integration of gymnastics in traditional activities, connected with a “longue durée” vision of the Swiss society and participating in a new process of “inventing a tradition” (Hobsbawm and Ranger, 1983) or in contrast with its neighbors' identity (Holenstein, 2018, p. 237). This new regulation aimed to establish the full national dimension of gymnastics (“die Leibesübung nationaler zu machen”) (Niggeler, 1859, p. 1), as pointed out in 1859, with a text published in the *Turnzeitung*, the official organ of the SFG. Johannes Niggeler was one of the pioneers of gymnastics in Switzerland, who had already worked to introduce the practice in several cities and who was to become the first long-standing central president of the SFG between 1870 and 1875 (Meier, 1957).

The man who would later be designated as the *Turnvater* of Swiss gymnastics insisted on the federative and liberal virtues of this “national gymnastics practice” for young men from all over the country, which also meant that he was sometimes banned by the authorities, for example in Bern when a new conservative government was elected in the early 1850's (Meier, 1957, p. 10–12), and forced to leave for La Chaux-de-Fonds. A couple of years later, when conservative Catholics were gaining more and more political power in Switzerland and their ultramontane branch asserted itself in the context of the “Kulturkampf” (Altermatt, 1995, p. 55–56) in line with the Vatican's desire to promote traditional values in an industrial society considered as morally gangrenous (Meuwly, 2013, p. 86), Niggeler maintained his vision:

“There is no better opportunity to collect young people and men from all regions of our homeland under the banner of freedom and progress, to unite them and to strengthen them against emerging reactionary and ultramontane aspirations than the Swiss Federal Gymnastics Society and its Festivals.” (Niggeler, 1859, p. 3)

In the first decade of the twentieth century, the support for progress and the promotion of patriotism based on youth must be reconsidered in the light of the affirmation of nationalism and of a “conservative overthrow” that took place at the head of the SFG from the late nineteenth century onwards. The phenomenon has to be seen against the general rise of conservatism in Switzerland since the 1870's (Jost, 1992, p. 31), even in Protestant and liberal-radical circles, the traditional breeding ground for gymnasts. This reactionary wave saw the advent of a new political stream, called the “new right,” during the last decade of the nineteenth century, that notably has antisocialism in common with the radical current (Meuwly, 2013, p. 125). This “new right”, now politically organized, was symbolized by the entry of the first conservative Catholic, Josef Zemp, into the Federal Council in 1891.

The SFG's 1907 anniversary book was thus an opportunity to praise the traditional value of work and to underline the lightweight nature of the youth of the time, against which gymnastics was presented as a cure:

“Let the Homeland call, the gymnast will do his duty. But the good gymnast won't wait until the end of the war; everyday life already gives him the opportunity to do so. Our economic independence, to which our political freedom is closely linked, is increasingly called into question every year, and loyalty to the profession and conscientiousness of work are necessary weapons as guns and cannons. […] In the interior of the country, the danger of indifference to public affairs and the jaded attitude of many young people threatens.” (Spühler et al., 1907, p. 121)

To take up Jacques Defrance's idea, subsequently explored in more depth by Grégory Quin, of the dependence of the field of physical activities on other fields (education, politics and religion) in France at the end of the nineteenth century (Defrance, 1995, p. 18; Quin, 2011), the majority of Swiss local gymnastics clubs incorporated in their objectives the patriotic and youth training goals proclaimed by the SFG, whose active involvement in physical education and preparation for military service as early as 1874 was now established.

It is also worth mentioning that in the last decades of the nineteenth century, Switzerland saw the emergence of workers' and confessional Catholic gymnastics clubs, along with new “modern sport” organizations, all creating competition for the SFG. From the 1860's to the 1880's the foundation of the first mountaineering, rowing, cycling (Jost, 1998, p. 33–34) and football clubs (Berthoud et al., 2016; Koller, 2016; Gogniat, 2018) in the country, combined the apogee of the development of gymnastics clubs with the foundation of the first clubs formed around emerging sports practices, and then their parastatal role can be seen as an advantage toward new organizations.

Gymnastics circles generally took a dim view of the arrival of new sports while trying gradually to integrate them into their traditional panel of activities (Bussard, 2007). The downward trend from 1900 onward in the number of gymnastics clubs founded probably illustrates the new competition within the “field,” around some brand-new physical activities, perhaps seen as more attractive to youth. On the other hand, the process of institutionalization of Swiss gymnastics had probably matured and was stabilizing at the beginning of the twentieth century as it reached almost all the regions, even beyond the biggest cities and especially in new suburban areas.

Thus, the overall analysis in this chapter supports the hypothesis that gymnastics in Switzerland is above all “an essentially urban, industrial and Protestant phenomenon” (Marcacci, 2019, p. 141), which was based on the involvement of young people, and has echoes in the processes of dissemination of modern sports. Thus, those elements also interestingly challenge some classical interpretations about the alleged “ideological opposition” between gymnastics and modern sports, inviting us to deepen our analysis.

## Conclusion

Beyond evidence relating to the participation of gymnasts and gymnastics leaders in the construction of “modern Switzerland” in the second half of the nineteenth century, the ambition of the gymnastics world might also be understood as deeply political, while it is a clear parallel project to the mandatory gymnastics classes provided in schools from 1874. Having 16 as the age for joining the gymnastics clubs also made sense, when compulsory school gymnastics involved schoolboys aged from 10 to 15 (Burgener, 1952, p. 129). Later on, after the age of 20, young men could move on to other commitments in society, some of them being part of the boom of the *Société Suisse des Carabiniers*, whose numbers exploded, having 1,432 clubs and almost 70,000 members in 1900—from 435 clubs and little more than 20,000 members in 1890—(Gamma, 1924, p. 133), then joining other kinds of associations and social organizations, but also increasingly being able to stay in the “Männerturnverein” in a life-long commitment.

In some ways, the elements we have presented concerning the “youth” of gymnastics club members should be put in perspective by the fact that the turnover is also significant throughout the period we have studied. It is thus possible to consider the SFG clubs as the primary places for young people to pursue their physical and “national” training after leaving school and before entering military service. The growing number of clubs and members also allowed the emergence of a more proper patriotic project, while the numbers (690 clubs and 56,661 members) in 1907 rose to new heights (1,030 clubs and 89,222 members) in 1920 (SFG, Census for 1920). If this should also lead to a deeper analysis of the changing views of the leaders—the members of the Central Committee of the SFG (Vonnard et al., 2019)—the numbers are also to be understood as pre-conditions for the “autonomization” of the SFG within a field of sporting activities and they call into question the socio-political processes which defined Switzerland throughout the period of the First World War.

Interestingly, the book published for the 100th anniversary of the SFG ended with a table showing the creation of gymnastics clubs year by year, canton by canton, making clear the rise over the two decades from 1880 to 1900, especially between Bern, Aargau, Zurich, and St. Gallen. It also showed very accurately how the period from 1910 onwards corresponds to what can then be described as a second wave for gymnastics (Société Fédérale de Gymnastique, 1932, Annex 3). If the decision to promote youth under the age of 16 had to wait until 1917, several local gymnastics clubs—especially in towns—were already accepting gymnasts under 16 from the end of the 1880's onwards (Bussard, 2007, p. 153). As shown in the 1932 commemorative book, the geography of the new “Jungturnen” is slightly different from what we have observed, having Bern and Zurich as main cantons (with respectively 79 and 59 groups of young gymnasts), but Vaud or Neuchâtel also being part of this process (47 and 25 groups), while Aargau or Solothurn seemed to remain apart (11 and 17 groups) (Société Fédérale de Gymnastique, 1932, p. 214–215), making bigger cities probably even more important then. Thus data for this period should be analyzed more attentively and also replaced in a European history of the development of physical practices (Brühwiler, 2017), leaving the door open for further research on the interwar period, but also on the role of the First World War in this so-called second wave, between an emerging competition with modern sports (Bussard, 2007, p. 187–217) or new forms of physical activities, contemporary interrogations around the internationalization of sport (Quin et al., 2019) and around the early role played by the women's sub-organization for the national extension of gymnastics in Switzerland (Quin, 2015).

## Data Availability Statement

The original contributions presented in the study are included in the article/supplementary material, further inquiries can be directed to the corresponding author/s.

## Author Contributions

All authors were equally involved in the research, analysis, and interpretation around the topic of the article.

## Conflict of Interest

The authors declare that the research was conducted in the absence of any commercial or financial relationships that could be construed as a potential conflict of interest.
